# Characterization of Unique Small RNA Populations from Rice Grain

**DOI:** 10.1371/journal.pone.0002871

**Published:** 2008-08-06

**Authors:** Sara E. Heisel, Yuanji Zhang, Edwards Allen, Liang Guo, Tracey L. Reynolds, Xiao Yang, David Kovalic, James K. Roberts

**Affiliations:** 1 Monsanto Company, Chesterfield, Missouri, United States of America; Cairo University, Egypt

## Abstract

Small RNAs (∼20 to 24 nucleotides) function as naturally occurring molecules critical in developmental pathways in plants and animals [1], [2]. Here we analyze small RNA populations from mature rice grain and seedlings by pyrosequencing. Using a clustering algorithm to locate regions producing small RNAs, we classified hotspots of small RNA generation within the genome. Hotspots here are defined as 1 kb regions within which small RNAs are significantly overproduced relative to the rest of the genome. Hotspots were identified to facilitate characterization of different categories of small RNA regulatory elements. Included in the hotspots, we found known members of 23 miRNA families representing 92 genes, one *trans* acting siRNA (ta-siRNA) gene, novel siRNA-generating coding genes and phased siRNA generating genes. Interestingly, over 20% of the small RNA population in grain came from a single foldback structure, which generated eight phased 21-nt siRNAs. This is reminiscent of a newly arising miRNA derived from duplication of progenitor genes [3], [4]. Our results provide data identifying distinct populations of small RNAs, including phased small RNAs, in mature grain to facilitate characterization of small regulatory RNA expression in monocot species.

## Introduction

Rice is one of the world's most important food crops, as it is produced in over 100 countries and is a staple food for half of the world's population (OECD, 2004). In addition to the importance of rice as a food staple, its genetic synteny, ease of transformation and an assembled genome [5] make it a model system for the study of cereal grasses. The importance of small interfering RNAs (siRNAs) and microRNAs (miRNAs) in plant developmental regulation have made the investigation of small RNA populations an important aspect in understanding the regulation of higher plant genomes and processes. Classes of small RNAs including miRNAs, ta-siRNAs, and nat-siRNAs regulate important developmental and physiological processes in land plants and most regulatory small RNAs and target genes are conserved among higher plants [1], [2], [6]. In general, these classes of regulatory RNAs suppress gene expression by inhibition of translation or destabilization of target mRNAs in *trans*. miRNAs and ta-siRNAs are derived from distinct transcriptional units that either form internal foldback structures or recruit a specific RNA-dependent RNA polymerase (RdRP) RDR6. Additional endogenous siRNAs often associated with repetitive elements have also been characterized that are processed from long double stranded RNA (dsRNA), and function to silence transcription in *cis* through modification of the chromatin state.

As in-depth annotation and functional gene network relationships are developed for rice, the analysis of small RNA expression will facilitate a deeper understanding of these relationships. An extensive and diverse population of small RNAs have been identified in Arabidopsis and rice utilizing high throughput sequencing methods [3], [7]–[14], however only developing or stressed tissues were evaluated [15]–[17]. In an attempt to gain a broader understanding of gene expression in rice grain, we characterized small RNA populations from *Oryza sativa* spp. *japonica* cv. Nipponbare utilizing a deep sequencing approach. As approximately 70% of all human food consumption is derived from seeds [18] it is important to understand the role of small RNAs in seed development given the known roles of miRNAs in organ identity, morphogenesis, and polarity in actively growing tissues. Seed development is a highly regulated and coordinated process involving deposition of seed storage reserves, novel morphogenic events to define the embryo and endosperm, and maturation drying and quiescence. Grain in the dormant state is typically characterized by little or no active translation, given this and the known roles of miRNAs in developing tissues only actively growing tissues have previously been analyzed in depth. The regulatory role of small RNAs in repression of gene expression could potentially provide an important mechanism in establishing or maintaining the dormant state in grain or altering storage reserves. In this study, we have initiated the sequencing of the small RNA population from rice grain and seedlings to determine the abundance and role of miRNAs and other small RNAs in both dormant and growing tissues.

## Results

### Deep sequencing of rice grain and seedling small RNA populations

Small RNA libraries were constructed from three pools of mature, dormant rice grain and three-week post germination seedlings utilizing RNA-adapter mediated ligation [19], [20]. Each of the four libraries were independently sequenced using high-throughput pyrosequencing [21]. We obtained a total of 679,146 sequences from the three rice grain libraries and 257,394 from one rice seedling library (Table 1). Greater than 41% of these sequences represent unique reads. Grain and seedling small RNAs were similar in size distributions with distinct peaks at 21- and 24-nt (Figure S1). Consistent with previous reports, the 21-nt small RNAs comprised many redundant reads, whereas the 24-nt class comprised primarily unique or low abundance reads. In seedling, greater than 30% of the ∼21-nt redundant reads matched to known miRNAs. Despite a similar distribution of ∼21-nt redundant sequences in grain, less than 5% could be accounted for by previously characterized miRNAs. The majority of these small RNAs were from non-repetitive elements, suggesting either many novel grain specific miRNAs or alternative siRNA generating loci such as nat- or ta-siRNAs. These data represent the first large scale sequencing of small RNAs from mature rice grain and an opportunity to assess the role of small RNAs in grain.

**Table 1:** Genome and transcriptome matches to rice small RNA sequences.

	Size (MB)	match^a^			match^a^ percentage (%)		
		Grain	Seedling	Total	Grain	Seedling	Total
**Total small RNAs**	n/a	679,146	257,394	936,540	n/a	n/a	n/a
**Unique small RNAs**	n/a	285,873	108,485	386,633	100	100	100
**Perfect matches to genomes**							
***Oryza sativa***	378	242,442	98,747	334,012	84.81	91.02	86.39
***Populus trichocarpa***	427	14,521	3,535	16,374	5.08	3.26	4.24
***Arabidopsis thaliana***	120	13,288	3,079	14,820	4.65	2.84	3.83
***Chlamydomonas reinhardtii***	105	5,876	961	6,280	2.06	0.89	1.62
*Homo sapien*	2,881	4,753	1,325	5,753	1.66	1.22	1.49
*Homo sapien* b	1,474	1,736	664	2,320	0.61	0.61	0.60
***Caenorhabditis elegans***	100	1,730	345	1,898	0.61	0.32	0.49
***Drosophila melanogaster***	118	621	162	752	0.22	0.15	0.19
***Magnaporthe grisea***	39	1,113	187	1,181	0.39	0.17	0.31
***Giardia lamblia***	11	539	107	569	0.19	0.10	0.15
**Perfect matches to transcriptomes** c							
*Oryza sativa*	104	37,616	10,050	46,565	13.16	9.26	12.04
*Oryza sativa (both strands)*	104	92,392	22,545	110,665	32.32	20.78	28.62
*Homo sapien*	105	256	66	300	0.09	0.06	0.08
*Drosophila melanogaster*	45	119	18	132	0.04	0.02	0.03
**Transcript matches in complementary to small RNAs**							
*Oryza sativa*	104	25,064	13,789	26,892	39.89	21.94	42.80
*Homo sapien*	105	1,647	85	1,704	3.95	0.20	4.08
**Transcript matches in sense strand to small RNAs**							
*Oryza sativa*	104	26,590	15,348	29,179	42.32	24.42	46.44
*Homo sapien*	105	1,792	133	1,867	4.29	0.32	4.47
**Transcript matches in both strands to small RNAs**							
*Oryza sativa*	104	18,978	10,108	20,763	30.21	16.09	33.05
*Homo sapien*	105	1,187	1	1,193	2.84	0.00	2.86

a Defined as perfect match to the entire small RNA.

b Repeats were masked by RepeatMasker and Tandem Repeats Finder (with period of 12 or less).

c Only complementary matches were considered except noted.

### Conservation of small RNAs in plants and animals

To contribute to our understanding of small RNA function and conservation, we compared rice small RNA sequences to genomes of species representing important lineages throughout evolution. We found the highest conservation among plant species with a lesser percentage similarity among distantly related species. For example, there are 13,288 matches between the rice grain small RNA and *Arabidopsis thaliana* genome and 621 between the rice grain small RNA and *Drosophila melanogaster* genome. As previously reported [22] there is little conservation of small RNAs between rice and any animal species tested (Table 1). Small RNAs conserved among distantly related non-animal species were generally low abundance sequences, whereas the highly abundant miRNAs were conserved among plants. While more small RNAs, primarily miRNAs, are conserved among plants, many small RNAs were also found with perfect homology to sequences in human and other animal transcriptomes (Table 1). The majority of rice small RNAs matched to unannotated intergenic regions, with only 29% matching to the rice transcriptome, including 12% to the complement. Sequences unmatched to the rice genome were likely a result of sequencing errors or from regions of the rice genome that remain unmapped. Here, we have not removed small RNAs matching to rRNAs, tRNAs, or sn/snoRNAs from the analysis as these are unlikely to be mRNA degradation products due to the requirement for a 5′ phosphate group in the cloning protocol. However, because these small RNAs could have multiple genomic origins, we have normalized to genome copy number to help remove this potential error. We have also chosen to keep these small RNAs in the analysis group, as interesting new classes of small RNAs from similar repeat regions have been reported (piwi-RNAs) and from alternative size classes [23].

### Categorization of small RNA populations through hotspot determination

**Figure 1:**
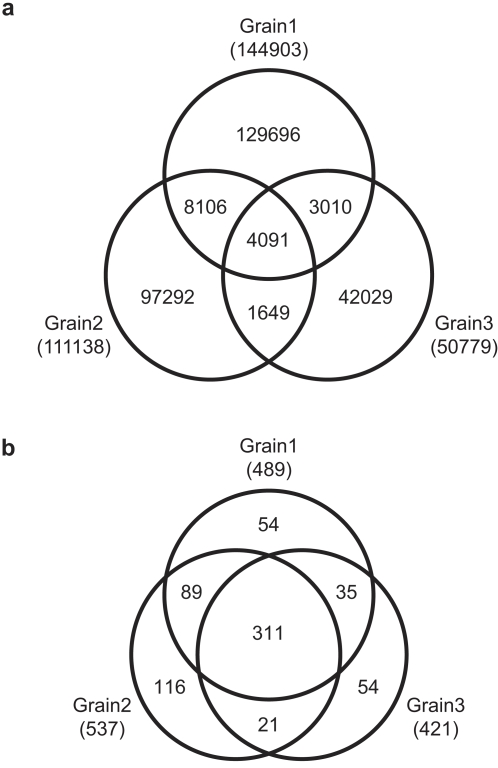
Overlap among small RNA sequences and hot spots. Venn diagrams illustrate overlap among rice grain replicate libraries (a) unique small RNAs or (b) 1-kb clustered hot spots (*P*<1E-50). The total number of unique sequences or hotspots from each replicate is in parentheses.

The three replicates of rice grain libraries allow us to estimate expression and assess the quality and coverage of each sequencing reaction. Amongst the three replicates, we obtained 285,873 unique sequences, however little overlap was observed among these sequences in independent libraries. We found only ∼1.4% of unique sequences were shared among all three replicates, and only ∼5.9% between at least two replicates (Figure 1a). This illustrates that despite the high number of sequences obtained; the endogenous rice grain small RNA population is far greater than captured by our sequencing effort.

While miRNAs represent a significant portion of the total number of sequences obtained, in comparison they represent a minor component of the unique small RNA population. In contrast, small RNAs from repetitive loci are disperse and may account for the low overlap among unique sequences from replicate libraries [10]. To determine if clustering small RNAs from specific loci would result in greater overlap of replicates, we calculated hotspots individually for each grain library replicate. Hotspots were identified by dividing the genome into 1-kb bins and small RNA abundance was calculated for each strand of the genome separately. We calculated a *P* value for each bin and defined hotspots as *P*<E-50 (see Methods S1). Of the 551,274 sequences from rice grain that mapped to the genome, 53.2% were captured in 680 clusters representing less than 0.1% of the rice genome. In contrast to unique sequences, 67% of hotspots were represented in more than one replicate (Figure 1). These results are similar to reports in Arabidopsis, in which little similarity was observed among unique small RNA sequences yet a greater overlap was observed among clustered small RNAs [10]. For further categorization of small RNA populations, we utilized hotspot cluster determination to facilitate identification of abundant, unique miRNA loci, and loci from which many low abundance sequences are derived.

**Figure 2:**
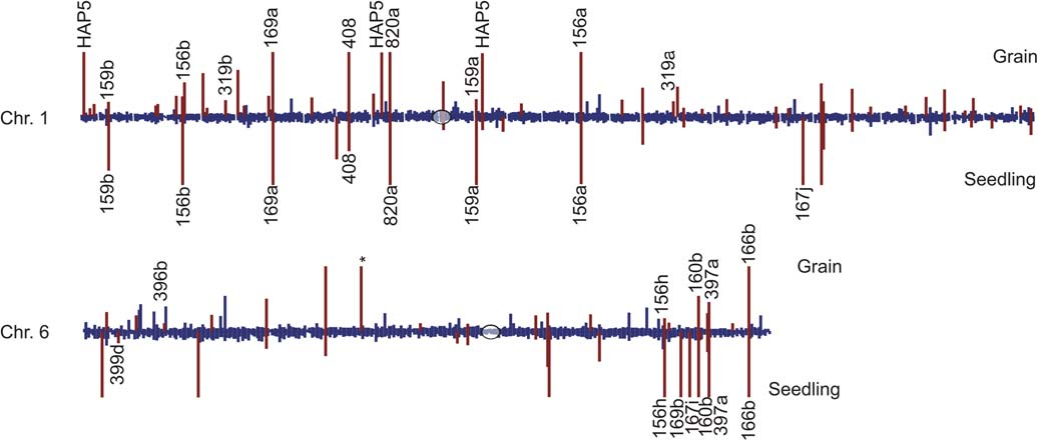
Small RNA expression from rice grain and seedling libraries across Chromosome 1 and Chromosome 6. Abundance was normalized by dividing the small RNA tpq by the number of perfect matches in the rice genome. For rice grain, the average tpq of the three libraries was used. Centromere position is indicated by a circle and bins containing small RNA hotspots are indicated in red. Each bar represents a 100 kb bin. A ceiling of 500 transcripts per quarter million (tpq) was used.

We examined the distribution of small RNAs by plotting abundance across the twelve rice chromosomes, calculated as transcripts per quarter million sequences (tpq) (Figure 2 and Figure S2). Regions of high small RNA expression around centromeres have been reported for Arabidopsis leaf tissue [10], [13]. In contrast, we generally did not observe a concentration of small RNAs in centromeric and pericentromeric regions using repeat-normalized expression. Rather, expression was localized to specific regions across the rice chromosomes, indicating the majority of the rice small RNA population derives from a small number of highly expressed loci. In many cases, the regions of highest localized expression mapped to known miRNA loci. Grain and seedling libraries showed distinct, tissue-specific expression patterns, with more miRNA-associated hotspots in seedlings. The higher number of miRNA hotspots is consistent with known roles for miRNAs in regulating development in plants [1].

**Figure 3:**
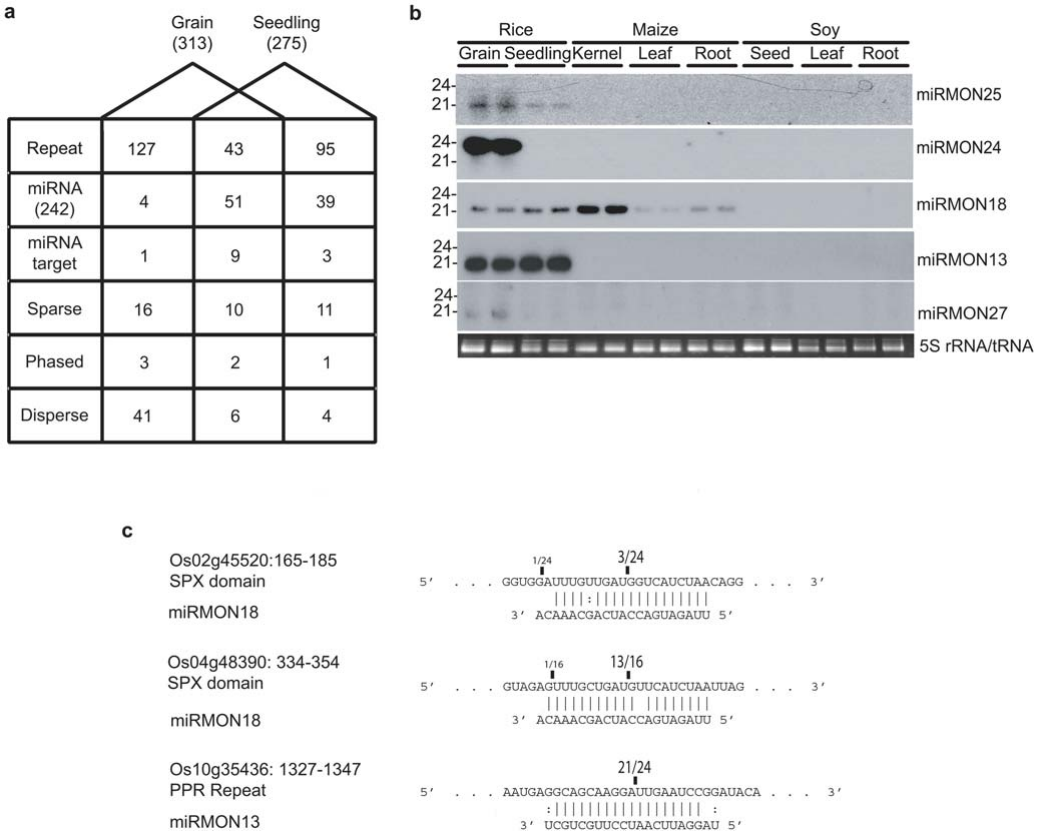
Characterization of rice hotspot small RNAs. (a) Classification of small RNA hotspots in rice grain and seedling small RNA libraries. Sparse hotspots contain one or two major peaks; disperse hotspots have more than two siRNA peaks; phased hotspots had a p-score >0.87. (b) Low molecular weight RNA blot analysis of rice miRNAs identified from hotspots in rice, maize, and soy. (c) Validation of predicted targets for 3 novel miRNAs. Positions of the dominant 5′ RACE products (sequences with 5′ ends at position/total sequences for 5′ ends) are indicated. Bolded nucleotide indicates predicted cleavage site.

Small RNAs from hotspots of seedling and grain libraries were categorized into repetitive elements, miRNAs, miRNA targets, sparse hotspots, phased hotspots such as ta-siRNAs, and disperse hotspots (Figure 3a). Phased hotspots were defined as those regions from which small RNAs were generated in a 21-nt register, while disperse hotspots were those regions of the genome from which many small RNAs were generated with no particular phasing or clustering. The reasons for evaluating hotspots were twofold; first they represent the population of small RNAs most likely involved in modulating gene expression in these tissues, and second the potential for incorrectly characterizing small RNAs decreases when low abundance sequences are not considered [1]. Furthermore, in the absence of a panel of RNAi pathway gene knockouts, such as is available in Arabidopsis, hotspot characterization helps to elucidate the functional classification of a given small RNA because the neighboring small RNAs are included in the analysis. For example, miRNA genes show two characteristic peaks of abundance, often located within a short distance of each other, with the miRNA sequence several times more abundant than the partially complementary miRNA* sequence. In this way, important regulatory elements such as miRNA and trans-acting siRNA loci can be found, although potentially at the expense of a number of false negatives due to the stringent requirements on abundance.

The unique small RNA sequences from grain and seedling were significantly different, which could reflect either lack of sequence coverage to fully capture the complexity of the small RNA population, or regions of small RNA expression unique to each tissue. To subvert this obstacle we evaluated genomic regions by categorizing 1 Kb regions (as described above), rather than individual small RNA sequences. This changed the overlap of the three rice grain replicates to 67% for 1 Kb bins over 1.4% overlap that was found when unique sequences were compared (Figure 1). For analysis of the three rice grain library replicates, we used the average normalized tpq. Using a cutoff of *P*<E-50 for hotspots resulted in a combined total of 498 small RNA clusters in grain and seedling (Table S2). The largest class of hotspots contained repetitive elements, such as transposons, ribosomal DNA, and tRNA genes. The second largest class was from MIRNA genes for which we captured 93 loci[24]; thirteen miRNA targets were also captured by similarity to truncated miRNAs. Consistent with the role of miRNAs in development and morphology, we detected more miRNA loci hotspots specific to seedlings (39) when compared to grain (4). The remaining hotspots were separated into sparse clusters associated with one or two specific peaks, phased, and disperse hotspots. Each of these categories was then analyzed for novel miRNAs, ta-siRNAs, and siRNA regulated genes, respectively.

### Identification of novel miRNAs by hotspot determination

Discovery efforts in Arabidopsis and rice have used abundant clusters containing only a small number of unique siRNAs as a starting point for prediction of novel miRNAs [3], [10], [12]. Following this rationale, we utilized sparse hotspots to search for new miRNAs. We propose that with extensive sequencing the propensity for false positive miRNA identification increases dramatically if low abundance siRNAs are considered. For example, using criteria derived from known miRNAs [1], [25] we predicted 840 candidate miRNAs from 1072 loci that form miRNA-like precursors. Potentially, many of these miRNA candidates represent species specific miRNA-like genes arising from recent duplication of progenitor sequences [3], [4]. In contrast, among sparse hotspots from grain and seedlings only five loci were found to contain miRNA-like foldback structures. While many of the 1072 miRNA-like loci are likely to represent *bona fide* miRNAs, we chose to only analyze the five corresponding to sparse small RNA hotspots. Expression of putative miRNAs from sparse hotspots was confirmed by Northern blot analysis (Figure 3b). All miRNAs except miRMON18, also detected in maize, were specific to rice. Similarly, the vast majority of recently identified miRNAs in Arabidopsis were non-conserved [3], [12]. Based on criteria for prediction of known miRNAs [1], [25] from which we predicted 840 potential miRNAs, rice also appears to have a diverse set of non-conserved miRNAs, which appear as low abundance sequences.

### Prediction of target genes for rice miRNAs

We predicted targets of the five new miRNAs using a scoring system that penalizes weak pairing to the 5′ end of the miRNA to reduce false positive predictions [26]. The results of this analysis were compared to small RNA hotspot clusters within predicted protein coding genes, including those flagged for short 18-nt matches. We expect to find miRNA targets in small RNA hotspots because many miRNAs are highly similar to their target sequences [27] and due to the possibility of transitivity [28]. We found thirteen previously predicted miRNA targets among clusters, supporting this hypothesis (Table S2). In addition, we were able to identify the PPR repeat target (Os10g35436) of miRMON13 utilizing hotspot classification, which was later validated by 5′ RACE assay.

Predicted targets of new miRNAs included an SPX-domain (miRMON18), PPR repeats (miRMON13), and CACTA transposon (miRMON22) gene families (Table S4). A non-conserved miRNA, miR827, predicted to target SPX genes in Arabidopsis [3], [12] varies by two nucleotides from miRMON18. Targets Os02g45520 and Os04g48390 are most similar to a subclade of Arabidopsis SPX genes predicted to be targets of miR827 [29]. Targets, Os02g45520 and Os02g48390 (SPX genes) for miRMON18 and Os10g35436 (PPR) for miRMON13 were tested using a standard 5′ RACE analysis. Cleavage events at the predicted site, 10 nucleotides from the 5′ end of the small RNA, were detected for all three predicted targets (Figure 3c). Three week old seedling tissue was used for validation of targets. Due to the similarity between the mature miRNA and predicted targets, miRMON18 is most likely a related family member of miR827 found in monocot accessions. miRMON13 was predicted to target seven PPR genes from an orthologous clade of PPR genes that have spurned miRNAs and ta-siRNAs in Arabidopsis [3], [26], [27], [30]. Target predictions for the remaining three novel miRNAs (miRMON24, 25, and 27) were performed but consisted largely of unannotated regions of the genome. This could be due to a lack of transcribed data for rice grain or due to the rapid evolution of miRNA genes [3], [4]. There is also no public microarray data for rice grain and low coverage with EST data for this tissue. This makes evaluating the possible functions of these genes difficult. Genes predicted to be regulated by abundant or grain-specific small RNAs are, however good candidates for further functional characterization and evaluation of their involvement in grain development.

### Small RNA generating hotspots from rice grain include phased siRNAs and protein coding genes

In our small RNA libraries, miRNAs accounted for 33.0% of all seedling small RNAs, in comparison to only 2.4% from grain (Figure S1). Despite the difference in miRNA abundance, 21-nt small RNAs were essentially equivalent in abundance from both tissues (Figure S1). We hypothesize that alternative regulatory siRNAs, including ta-siRNAs and naturally occurring antisense siRNAs, (nat-siRNA) might account for the discrepancy in miRNA abundance between grain and seedling 21-nt siRNAs [31], [32]. To test this hypothesis, we determined phased hotspots by calculating phase uniqueness (p-score) based on ratio of abundance of in-phase siRNAs to out-of-phase siRNAs and fullness of each phase (see Methods S1). A single TAS3-like gene on Chromosome 3 was identified among phased hotspots (Table S2) [26]. Additional phased siRNAs from three hotspots on chromosomes 6 and 12 together accounted for ∼22% of the 21-nt small RNAs from rice grain. Unlike ta-siRNAs and nat-siRNAs, phased siRNAs from both loci were exclusively from one strand, suggesting an alternative origin.

**Figure 4:**
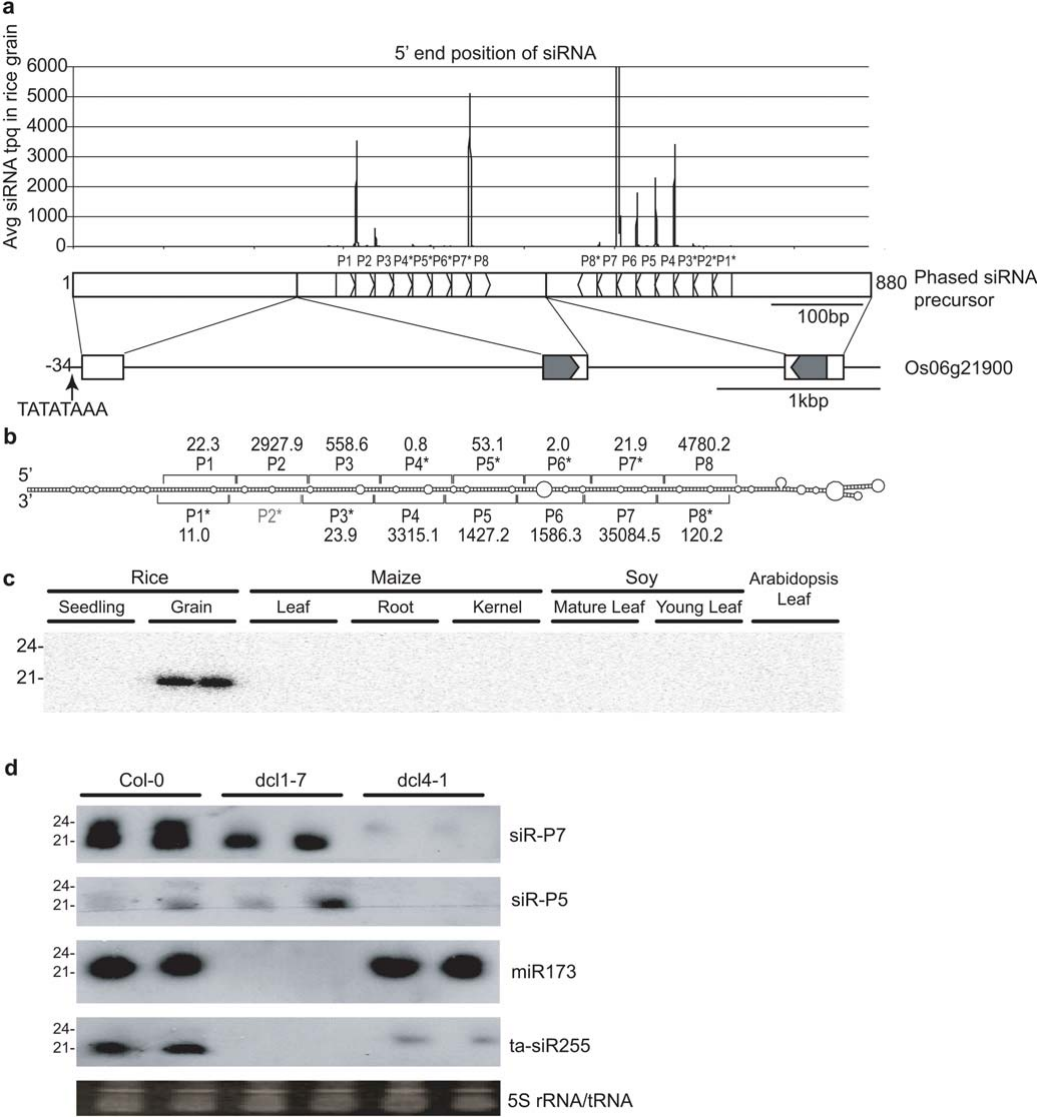
Phased siRNAs from the Os6g21900 locus. (a) Graph of 5′ position of siRNAs from the plus strand precursor RNA. Expression was capped at 6000 tpq. The abundant phases (P1–P8) and passenger strand siRNAs (P1*–P8*) are indicated. The locations of exons and introns are shown along the rice genomic sequence. (b) Predicted foldback structure formed by exons 2 and 3. Phasing is indicated by brackets and the tpq for each 21-nt phase is shown. (c) Low molecular weight RNA blot analysis of P7 siRNA expression. (d) Low molecular weight RNA blot of phased siRNAs from positions P5 and P7 in *A. thaliana* Col-0, *dcl1-7*, and *dcl4-1*. Expression of miR173 and ta-siRNA255 are shown as controls for canonical miRNAs and ta-siRNAs.

We chose to examine the locus on Chromosome 6 in detail, due to it generating siRNAs with the highest abundance, which were specific to rice grain. The phased siRNAs were distributed between two tightly clustered regions (Figure 4a). We cloned an 880-nt precursor that mapped to the Os6g21900 locus (see Data S1). Exons 2 and 3 form a long, imperfect foldback structure containing eight 21-nt phased siRNA duplexes, separated by an ∼1.2 kB intron (Figure 4b). No small RNAs were found that match exon I, nor were we able to identify a miRNA target sequence that could initiate ta-siRNA phasing [26], [33], [34]. RNA gel blot analysis of the most abundant phased siRNA confirmed expression specific to rice grain (Figure 4c). We were unable to detect either of the two most abundant phased siRNAs (P7 and P4) in seedling or other plant species tested. To determine whether the phased siRNAs are expressed in other tissues, we also compared the precursor to public EST collections and the rice MPSS database. All matched ESTs and MPSS signatures were from rice grain or six days post germination libraries. In addition, the passenger strand was also cloned for all phased 21-nt siRNAs (except P2), confirming siRNA biogenesis from the foldback structure.

We predicted that phased siRNAs would be processed by DCL4 due to the phased nature of 21-nt siRNAs, and would not require miRNA pathways because of the presence of a foldback structure in the Os06g21900 phased siRNA precursor RNA. To test this hypothesis, the full length cDNA from the Os06g21900 locus was transformed into *Arabidopsis thaliana* Col-0 ecotype, and mutants *dcl1-7* and *dcl4-1* diagnostic for miRNAs and ta-siRNAs, respectively. We detected P5 and P7 phased siRNAs in Col-0 and *dcl1-7* (Figure 4d). The abundant 21-nt phased siRNAs were absent in *dcl4-1*, replaced by faint 24-nt siRNAs, similar to what was observed for ta-siR255. Foldback structures with DCL4-dependent phased processing were observed for ASRP1729/miR822 and miR839, although in both cases the phasing was less precise than that seen for Os06g21900 [4], [12]. Furthermore recent reports [35] of DCL4-dependent generation of 21-nt small RNAs in rice suggest DCL4 plays a broader role than in Arabidopsis development and support our hypothesis that DCL4 can process phased small RNAs independent of an RdRP. Analysis of our data revealed an inverted repeat on chromosome 12 as a hotspot for generation of phased siRNAs. Small RNAs generated from this locus were identified in two previous reports [12], [35] as being dependent on DCL4. Together, these results confirm that phased siRNAs are processed through a distinctly different pathway than canonical miRNAs.

In Arabidopsis, miRNA-like genes have been characterized in which multiple phased ∼21-nt siRNAs are processed from a single foldback structure derived from duplication of progenitor genes [4], [12]. To test the possibility that this locus is a recently emergent miRNA-like gene, we compared the halves of the foldback sequences to protein coding genes in rice. Consistent with this hypothesis, we found significant similarity (E<10^−7^) to proton-dependent oligopeptide transporter (POT) genes (Figure S3). Phased siRNAs are reminiscent of miR163 in Arabidopsis in which two phases of siRNAs were sequenced, although only miR163 accumulates significantly [4], [36]. Unlike TAS loci in Arabidopsis that require RDR6 to generate dsRNA, phased siRNAs derive from an extended imperfect foldback structure. Given the high abundance of multiple, phased siRNAs from a single foldback precursor transcript and processing, we believe that these phased siRNAs represent a novel regulatory class present in rice grain.

**Figure 5:**
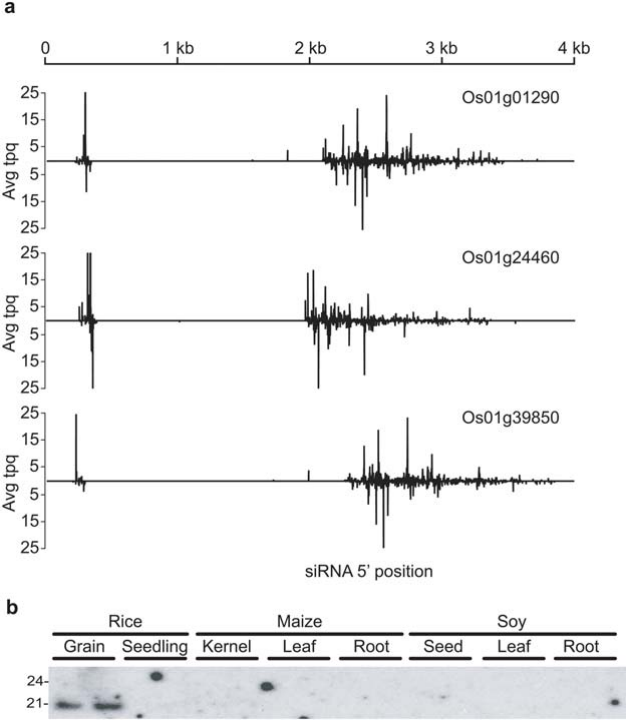
Disperse siRNAs associated with HAP5 genes. (a) Small RNA tpq is plotted as in Figure 4, with plus strand siRNAs above the x-axis, and minus strand below. Expression was capped at 25 tpq. (b) Low molecular weight RNA blot analysis of single siRNA.

In addition to the phased siRNAs found on Chromosome 6, we inspected three abundant disperse hotspots on Chromosome 1 from the grain libraries that map to HAP5 transcripts (Table S2). In contrast to phased siRNAs, individual siRNAs were in low abundance, confined to the transcribed region, and randomly distributed across the transcript (Figure 5). Based on similarity comparisons and secondary structure predictions, we found no evidence of inverted repeats at any of the three HAP5 loci on Chr 1 ((Os01g01290, Os01g24460, Os01g39850) (Table S2)). Upstream of each HAP5 gene there is an associated siRNA region, with weak phasing (p-score 0.691). Greater than 82% of the siRNAs from the three HAP5 hotspots were 21 to 22-nt, whereas siRNAs typically associated with disperse clusters are typically 24-nt [37]–[39]. A likely explanation for siRNA production would be bi-directional transcription forming nat-siRNAs [31]. We searched the MPSS database for signatures, and found signatures for expression only in the sense orientation in 6-day old seedlings [40]. There is little data available for mature rice grain, so it is possible that antisense transcription is responsible, although we favor a model in which an RdRP is recruited to the three HAP5 transcripts derived from Chromosome 1.

## Discussion

While our efforts in deep sequencing of rice grain and seedling tissue have revealed a number of unique elements in the regulatory small RNA family of rice, the functions of many of these remain to be elucidated. We have developed a method of hotspot determination to uniquely validate the class of small RNA in the absence of readily available mutants, which would lend themselves to characterization of small RNAs. Characterization in this manner allows for more rigorous analysis of small RNA data from sequenced genomes, despite the fact that it may produce false negatives, as indicated by the predicted 1072 pre-miRNAs based solely on foldback data. Using sparse hotspots as the defining criteria for miRNAs limited this set to five novel miRNAs, which were validated by Northern blot and predicted targets for two of the five novel miRNAs were validated by 5′ RACE analysis. Furthermore, we compared miRNAs from our large prediction set to those reported recently from wheat [41] and found 3 possible homologs with 4 to 5 nucleotide differences, but did not find any identical small RNAs. We verified 101 MIRNA genes in 27 families with high expression in mature grain and 3 week old seedlings by their presence in hotspot clusters.

Target prediction for the 5 novel miRNAs, revealed that miRMON13 targets a clade of PPR genes. The PPR family has expanded considerably in plants, comprising >450 genes in Arabidopsis and >600 in rice [42], [43]. Although it is not surprising to see miRNA genes derived from a rapidly expanding gene family, the preference to maintain suppression elements specific to a specific clade of PPR genes both in Arabidopsis and rice is curious. One possible model is that maintenance of proper gene dosage from this PPR clade is critical to plant fertility. Fertility restoration genes (Rf) have been found to contain PPR motifs, and mutations lead to cytoplasmic male sterility. Rapid evolution of miRNA and ta-siRNA genes from PPR progenitor genes may offer a mechanism to suppress aberrant or excess transcripts to prevent reduced fertility [3], [12].

In Arabidopsis, small RNAs principally of the 24-nt class, accumulated in pericentromeric regions [10], [13] whereas we observed a very different distribution in rice. Small RNA hotspots were more randomly distributed across the chromosomes, with 21-nt loci including miRNA and phased siRNA genes more readily apparent. This may be reflected in the tissues used for analysis, as epigenetic states should be established in mature grain and therefore accumulation of heterochromatin associated siRNAs is not required to the extent it would be in growing floral or vegetative tissues. Small RNA hotspot clusters accounted for over 53% and 49% of the total small RNA abundance in grain and seedling, respectively. In both tissues, clusters were confined to a very small proportion of the rice genome indicative of unique small RNA generating genes, many with unknown function. Many of these hotspots were not attributable to novel miRNAs or other characterized classes of small RNA generating loci; characterization of these genes is likely to reveal interesting and new mechanisms for gene regulation in plants.

A striking finding was the abundance of 21-nt siRNAs specific to rice grain that were not attributable to miRNAs. We identified hotspots expressing extraordinarily abundant 21-nt phased siRNAs (Os06g21900) or disperse ∼21-nt siRNAs (Chr. 1 HAP5 genes, Os01g01290) specific to dormant grain. Similar miRNA-like genes have been described in Arabidopsis [4], [12], from which multiple, phased 21-nt siRNAs dependent primarily on DCL4 are expressed. In contrast, the phased siRNA genes in rice showed a far stricter phasing (out-of-phase siRNAs did not accumulate), and expression of nearly all phases were equivalent to expression of conserved miRNAs. The phased siRNA precursor at Os06g21900 displays similarity to POT family nitrate transporters, consistent with a model in which miRNA genes derive from inverted duplication of progenitor genes [3], [4], [12].

In addition to abundant grain specific phased siRNAs, three HAP5 loci were found that generate an extraordinary number of 21-nt siRNAs across the length of the transcript. These transcripts could represent nat-siRNA loci, although we were unable to identify any antisense cDNAs or MPSS data indicating antisense PolII transcription. This could be due to the lack of available transcript/cDNA from mature rice grain. There is no evidence for inverted repeats from the transcribed regions at these loci, indicating that these (siRNAs) are unlikely to be DCL4 products Together with HAP2 and HAP3 proteins, HAP5 forms a ternary CCAAT transcription factor complex. This complex has known roles in controlling expression of seed storage protein genes in Arabidopsis [44], [45]. Our finding that, a subset of HAP5 genes generate such a large number of siRNAs in mature seed suggest post-transcriptional regulation of the HAP complex, potentially involved in modulating seed storage proteins. From the scope of all small RNA space, we observed that the reverse complementary match to the rice transcriptome is 40% over-represented for the unique grain small RNAs versus those from the seedling (Table 1. 13% vs. 9%). This over-representation of potential transcription repressors in grain might facilitate maintaining a general expression suppression state in the dormant grain stage. Further investigation into the roles of these siRNAs could lend insight into biology of seeds and the roles of these distinct populations of siRNAs.

All data will be deposited into NCBI GEO upon publication of this manuscript.

## Methods

### Plant materials and RNA isolation

*Oryza sativa* spp. *japonica* cv. Nipponbare was used for small RNA library construction and Northern blot analysis. Plant material for RNA isolation was obtained from dehulled mature grain and 23 day old seedlings planted from the same seed lot and grown in a greenhouse under non-stress conditions. RNA was isolated for Northern analysis from *Zea mays* var. LH244 leaves, roots (both at stage V6), and 32–39 day after pollination kernels; *Glycine max* var. A3525 trifoliate leaves, roots, and S3 to S5 seed; and *Arabidopsis thaliana* ecotype Columbia-0. Seed for *dcl1-*7 (CS3089) and *dcl4-1* (GK_106G05) were obtained from the Arabidopsis Biological Resource Center. *A. thaliana* was transformed with a CaMV 35S binary expression vector using the floral dip method, and events containing a single T-DNA insertion selected. Total RNA was isolated from plant tissues using TRIzol reagent (Invitrogen).

### Construction and computational analysis of small RNA libraries

Small RNA library construction was performed as described previously [20]. Three micrograms of each small RNA cDNA library was sent to 454 Life Sciences for sequencing. Small RNA inserts in the raw sequences were parsed by locating the 5′ and 3′ adaptors to obtain strand and position information by Perl scripts. Small RNAs of 18–26 nt were compared with genomes using BLAST (Table S1 for databases used). To compare small RNA expression from different libraries, abundance was calculated as transcripts per quarter million sequences (tpq) [8], [10]. Normalized abundance was determined as tpq divided by the number of perfect matches to the rice genome. To determine small RNA expression hotspots in the rice genome, each chromosome was divided into 1-kb bins and small RNAs were assigned to specific bins. In cases where small RNAs were divided between bins, the 5′ end of the small RNA was used to determine in which bin it should be placed. Normalized small RNA abundances with each bin were summed to give the total abundance of the bin.

### Statistical analyses

See Methods S1 for detailed description of statistical methods used in analysis.

### Small RNA blotting and hybridization

Five micrograms of total RNA was separated on a 17% PAGE-urea gel and blotted as previously described [4]. Complementary oligonucleotide probes specific to small RNA sequences were end labeled with *γ* ^32^P-ATP using OptiKinase (USB Corporation) [46]. Probe sequences are listed in Table S3. LNA probes were ordered for miRMON13 and miRMON27. Oligo probes for ta-siR255, miR173, and phased siRNAs in Arabidopsis were end-labeled with digoxygenin according to the manufacturer's recommendation (Roche). Probe sequences are listed in Table S3.

### Target Validation

Target Validation using a 5′ RACE assay was done with the GeneRacer Kit (Invitrogen). Poly(A) mRNA was isolated from 3 week old rice seedling tissue, ligated to the adaptor, converted to cDNA and PCR amplified, using gene-specific and adaptor-specific primers. PCR products were gel purified, cloned and sequenced. Successfully validated miRNA target gene-specific primer sequences are shown in Table S3.

## Supporting Information

Figure S1 (a) The majority of small RNAs matched to miRNAs, rRNA repeats, and transposable elements. Annotated miRNAs from miRBase accounted for 33.0% of seedling small RNAs and 2.4% from grain. (b) The size distribution of small RNA populations from grain and seedling were similar, with 21-nt small RNAs as the most abundant class, followed by the 24-nt class.
(0.08 MB AI)

Figure S2 Small RNA expression from grain and seedling libraries, calculated as transcripts per quarter million (tpq) across the twelve rice chromosomes. Abundance was normalized by dividing small RNA tpq by the number of perfect matches in the rice genome. For rice grain, the average tpq of the three libraries was used. Centromere position is indicated by a circle and bins containing small RNA hotspots are indicated in red. Each bar represents a 100 kb bin. A ceiling of 500 tpq was used. Location of phased siRNAs are indicated by an asterisk (*). The majority of rice or monocot specific miRNAs are represented by a single locus.
(0.55 MB PDF)

Figure S3 Alignment of the foldback structure regions of exon 3 and the reverse complement of exon 2 from Os06g21900 with putative progenitor genes. Conserved positions are highlighted as follows: 7/7 red, 5/7 or 6/7 orange, 4/7 yellow, 3/7 green (including at least one conserved base in Os06g21900 foldback).
(0.09 MB PDF)

Table S1 Sources of genome and transcriptome data.
(0.02 MB XLS)

Table S2 Small RNA hotspots from rice.
(0.10 MB XLS)

Table S3 Small RNA probes for Northern blot analysis & Target Validation
(0.02 MB XLS)

Table S4 Predicted Targets for miRMON13 and miRMON18.
(0.02 MB XLS)

Methods S1 Statistical methods used in hotspot determination.
(0.05 MB DOC)

Data S1 Sequence of phased siRNA precursor Os06g21900
(0.02 MB DOC)
