# Supplementary material for: Characterization of Unique Small RNA Populations from Rice Grain
Source: PLoS One. 2008 Aug 6;3(8):e2871. doi: 10.1371/journal.pone.0002871 (PMC2518513; doi:10.1371/journal.pone.0002871)
Supplement: Figure S1 — (a) The majority of small RNAs matched to miRNAs, rRNA repeats, and transposable elements. Annotated miRNAs from miRBase accounted for 33.0% of seedling small RNAs and 2.4% from grain. (b) The size distribution of small RNA populations from grain and seedling were similar, with 21-nt small RNAs as the most abundant class, followed by the 24-nt class. (0.08 MB AI) [file pone.0002871.s001.pdf]

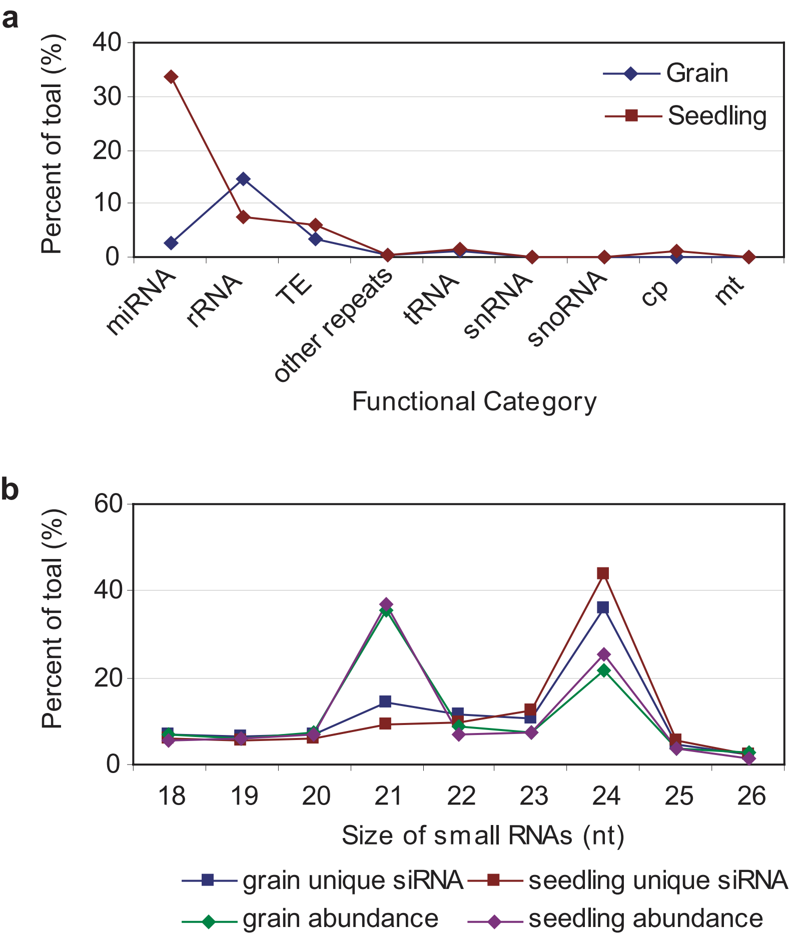

Figure S1. (a) The majority of small RNAs matched to miRNAs, rRNA repeats, and transposable elements. Annotated miRNAs from miRBase accounted for 33.0% of seedling small RNAs and 2.4% from grain. (b) The size distribution of small RNA populations from grain and seedling were similar, with 21-nt small RNAs as the most abundant class, followed by the 24-nt class.

Figure S1
