# Supplementary figures and images for: Characterization of Unique Small RNA Populations from Rice Grain
Source: PLoS One. 2008 Aug 6;3(8):e2871. doi: 10.1371/journal.pone.0002871 (PMC2518513; doi:10.1371/journal.pone.0002871)

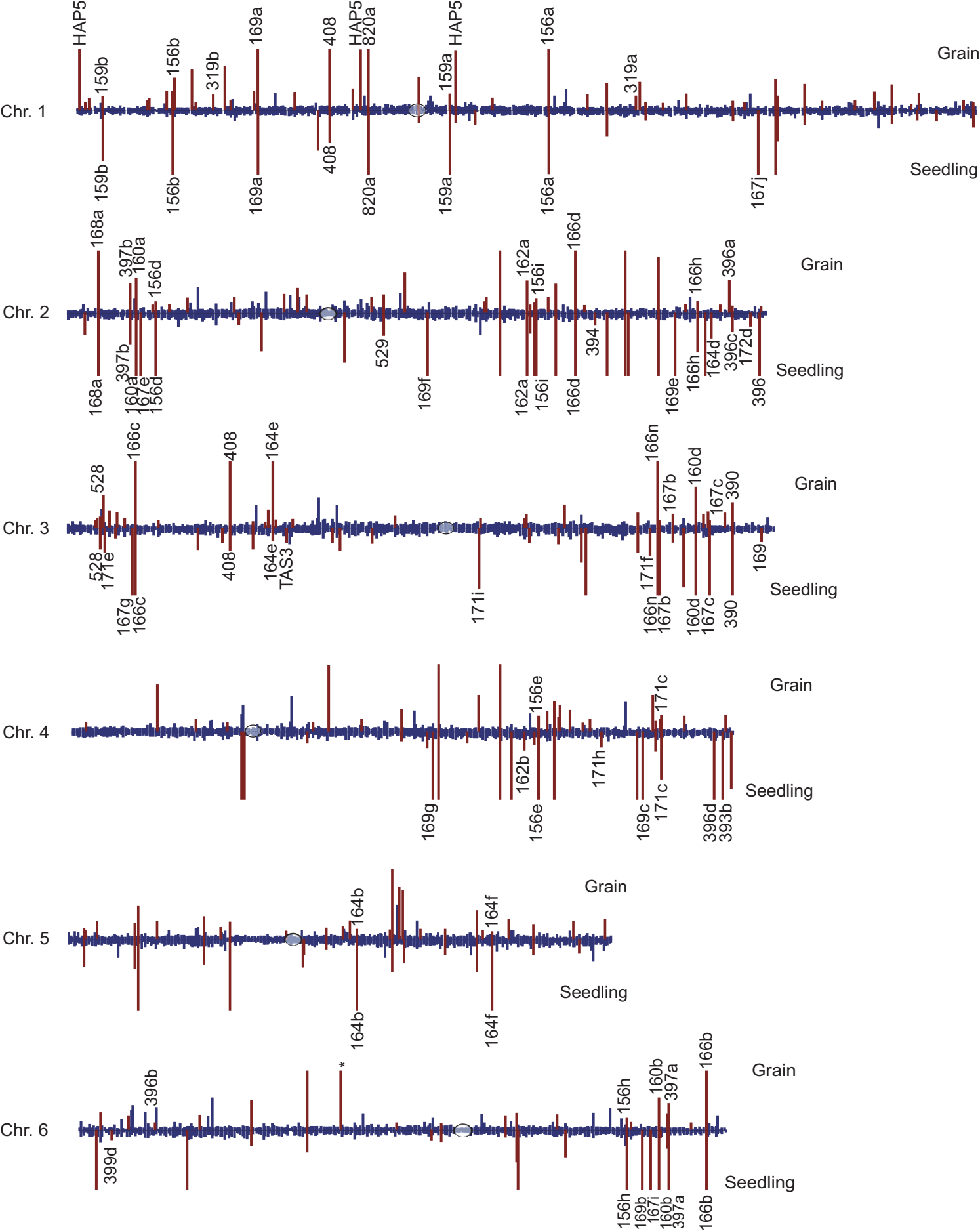

Figure S2

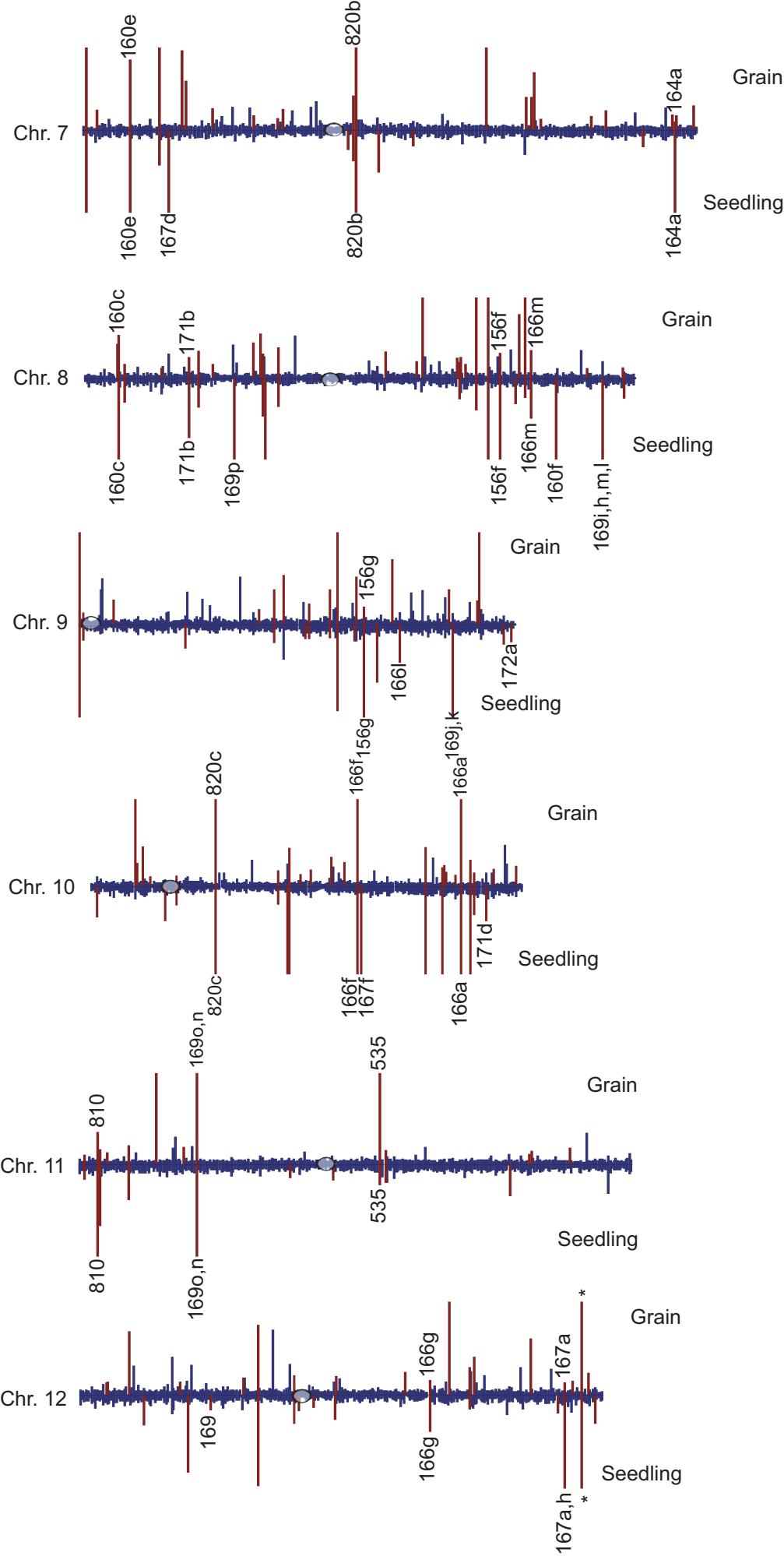

**Figure S2**

Supplement: Figure S2 — Small RNA expression from grain and seedling libraries, calculated as transcripts per quarter million (tpq) across the twelve rice chromosomes. Abundance was normalized by dividing small RNA tpq by the number of perfect matches in the rice genome. For rice grain, the average tpq of the three libraries was used. Centromere position is indicated by a circle and bins containing small RNA hotspots are indicated in red. Each bar represents a 100 kb bin. A ceiling of 500 tpq was used. Location of phased siRNAs are indicated by an asterisk (*). The majority of rice or monocot specific miRNAs are represented by a single locus. (0.55 MB PDF) [file pone.0002871.s002.pdf]
