# Supplementary material for: Characterization of Unique Small RNA Populations from Rice Grain
Source: PLoS One. 2008 Aug 6;3(8):e2871. doi: 10.1371/journal.pone.0002871 (PMC2518513; doi:10.1371/journal.pone.0002871)
Supplement: Figure S3 — Alignment of the foldback structure regions of exon 3 and the reverse complement of exon 2 from Os06g21900 with putative progenitor genes. Conserved positions are highlighted as follows: 7/7 red, 5/7 or 6/7 orange, 4/7 yellow, 3/7 green (including at least one conserved base in Os06g21900 foldback). (0.09 MB PDF) [file pone.0002871.s003.pdf]

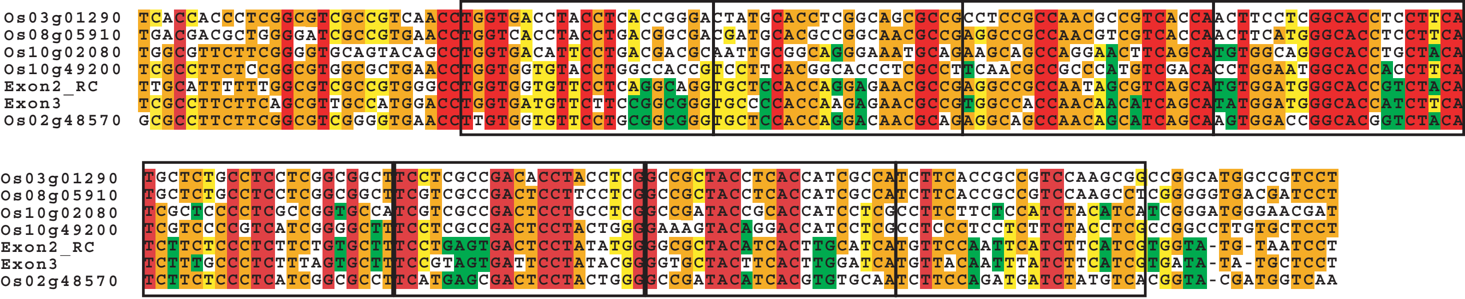

Figure S3. Alignment of the foldback structure regions of exon 3 and the reverse complement of exon 2 from Os06g21900 with putative progenitor genes. Conserved positions are highlighted as follows: 7/7 red, 5/7 or 6/7 orange, 4/7 yellow, 3/7 green (including at least one conserved base in Os06g21900 foldback).
