# Supplementary material for: Characterization of Unique Small RNA Populations from Rice Grain
Source: PLoS One. 2008 Aug 6;3(8):e2871. doi: 10.1371/journal.pone.0002871 (PMC2518513; doi:10.1371/journal.pone.0002871)
Supplement: Methods S1 — Statistical methods used in hotspot determination. (0.05 MB DOC) [file pone.0002871.s008.doc]

**Methods S1**

**Statistical analyses.**  The whole rice genome was first divided into 1-kb bins and small RNA abundance was computed for each strand of the genome separately. For each chromosome, because the histogram showed an empirical distribution very close to Poisson distribution (Supplementary Figure 4), we used such a distribution to describe the abundance of small RNA, as Poisson distribution was widely used to model count data (Spiegel, 1992). Using the observed abundance for all bins, an average abundance was estimated, and subsequently used to compute the statistical significance of observed abundance in each bin relative to the population averages, which ultimately defines a hotspot. Hotspots containing only 18 or 19 nt small RNA matches were excluded from further analysis. The following shows the statistical procedures on how to find hotspots for a given strand of the rice genome.

λ = 0.33

Figure S4. Histogram of bins with small RNA expression abundance for Watson strand of rice chromosome 1.

Assuming we have a chromosome with$K$bins with observed abundances$x_{1},\cdots,x_{K}$, respectively. The underlying population can be defined as a Poisson distribution with population mean$\lambda$, an unknown parameter to be estimated with observed data. The maximum likelihood estimate (MLE) of parameter $\lambda$ (Spiegel, 1992) can be obtained as

,

Supplemental Table 5 shows the estimate of average abundance for each strand of all 12 chromosomes. Once the population averages are estimated, a small RNA hotspot can be defined accordingly on a strand. For a bin with observed abundance$x_{i}$, we defined a significance level for the departure of observed abundance from the population average$\lambda$:

$P(X\geq x_{i})=\sum_{t=x_{i}}^{\infty} P_{p}(X=t)$, $i=1,2,\cdots,K.$

Where $P_{p}(X=t)$is probability density function of Poisson distribution with parameter. This equation defines the right-tail probability which measure how the observed abundance of a bin differs from the average abundance on a strand. Therefore, a hotspot is defined as a bin with its significance meeting certain criterion. The same process was applied to all chromosomes, and significance obtained for each bin. *P*-value cutoff 1E-50 was used to find hotspots along each strand.

Table S5. Average abundance (λ) of all bins for each strand of each rice chromosome

| Chromosome | Watson Strand | Crick Strand |
| --- | --- | --- |
| 1 | 0.33 | 0.24 |
| 2 | 0.49 | 0.5 |
| 3 | 0.23 | 0.2 |
| 4 | 0.21 | 0.22 |
| 5 | 0.17 | 0.21 |
| 6 | 0.2 | 0.23 |
| 7 | 0.22 | 0.27 |
| 8 | 0.3 | 0.25 |
| 9 | 0.26 | 0.23 |
| 10 | 0.29 | 0.26 |
| 11 | 0.15 | 0.22 |
| 12 | 0.43 | 0.21 |
| * outliers excluded. | |  |

Reference:

Spiegel, M. R. Theory and Problems of Probability and Statistics. New York: McGraw-Hill, pp. 111-112, 1992.
