# Supplementary material for: Characterization of Unique Small RNA Populations from Rice Grain
Source: PLoS One. 2008 Aug 6;3(8):e2871. doi: 10.1371/journal.pone.0002871 (PMC2518513; doi:10.1371/journal.pone.0002871)
Supplement: Data S1 — Sequence of phased siRNA precursor Os06g21900 (0.02 MB DOC) [file pone.0002871.s009.doc]

**Data S1**

Sequence of phased siRNA precursor Os06g21900

>LIB4833-001-R1-N1-G10

AATCTTATTCTACATATTTCTATCTTATATAGAACAACTAGCATAGCTCTCGTTGCCCAGCCAGGTTGCCCAGCCAGGTTGCCTGGTGCACAATGAGAGCTGGCTAGGGCGGACTCATTCTGCTGTTGGTGCCCAACGATGCTAGCTGCTACTCATACTAGTGAAGCCTGCCATGGTTCTGAGAAATTTTTGGATACTCCGCTGCGTAGATATGCACTAAAAGCTTGTATGTTTCGCTGACTACATACTATGGATATCACCTGTTTGACAAGAGAAGGATTACATACCACGATGAAGATGAATTGGAACATGATGCAAGTGATGTAGCGCCCCATATAGGAGTCACTCAGGAAAGCACAGAAGAGGGAGAAGATGTAGACGGTGCCCATCCACATGCTGACGCTATTGGCGGCCTCGGCGTTCTCCTGGTGGAGCACCTGCCTGAGGAACACCACCAGGCCCACGGCGACGCCAAAAAATGCAAAGTTGGCCAACACATAGCTCACTGCATCGTCAAGTAGAGCTGCTTAATCACTGAGGTATATACATTTAGTTCGCCTTCTTCAGCGTTGCCATGGACCTGGTGATGTTCTTCCGGCGGGTGCCCCACCAAGAGAACGCCGTGGCCACCAACAACATCAGCATATGGATGGGCACCATCTTCATCTTTGCCCTCTTTAGTGCTTTCCGTAGTGATTCCTATACGGGGTGCTACTTCACTTGGATCATGTTACAATTTATCTTCATCGTGATATATGCTCCTTCTGTTCTCACATAGGTGATATCTTAAAATGTATGAGGCATATATACTTTCTACCTAATATTATAAAGTATATGCCTCTATATAGATCAAATAAAGCAGAAAAGTCATTGTTATTACAAAAAAAAAAAAAAAAAA
